# Supplementary figures and images for: Comprehensive pathway-related genes signature for prognosis and recurrence of ovarian cancer
Source: PeerJ. 2020 Dec 1;8:e10437. doi: 10.7717/peerj.10437 (PMC7718801; doi:10.7717/peerj.10437)

A

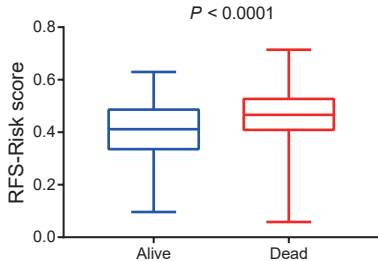

B

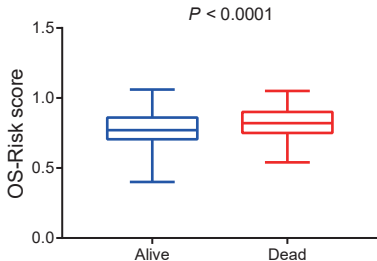

Supplement: Supplemental Information 1 [file peerj-08-10437-s001.pdf]

ROC curve

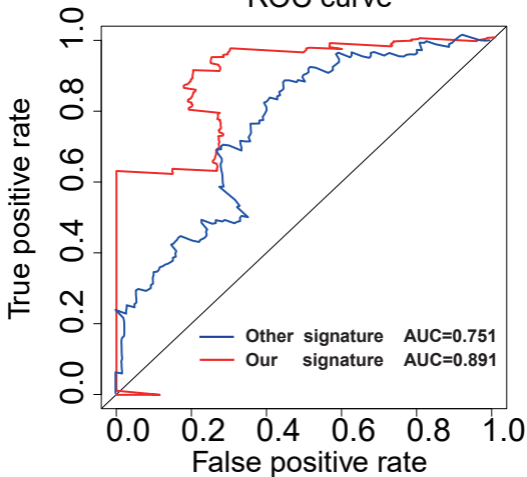

Supplement: Supplemental Information 3 [file peerj-08-10437-s003.pdf]

# GO analysis

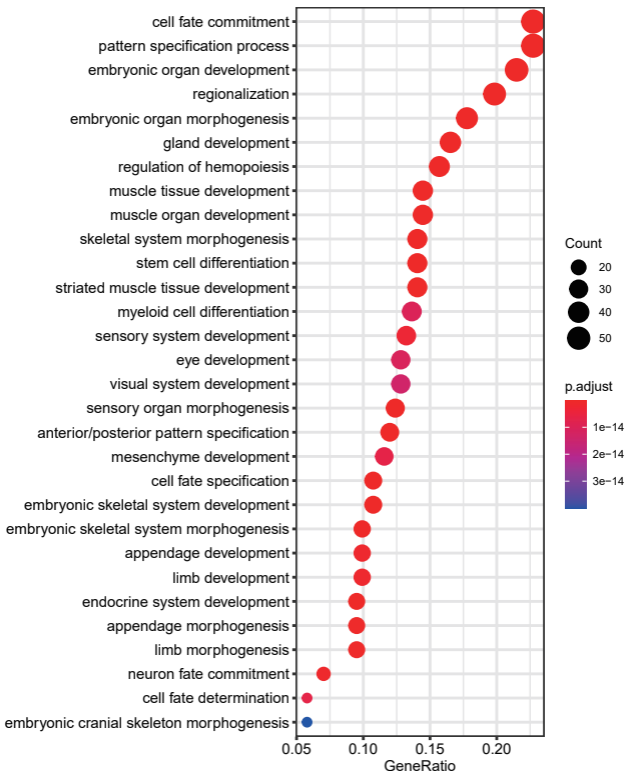

Supplement: Supplemental Information 4 [file peerj-08-10437-s004.pdf]
