# Supplementary material for: Comprehensive pathway-related genes signature for prognosis and recurrence of ovarian cancer
Source: PeerJ. 2020 Dec 1;8:e10437. doi: 10.7717/peerj.10437 (PMC7718801; doi:10.7717/peerj.10437)
Supplement: Supplemental Information 2 [file peerj-08-10437-s002.pdf]

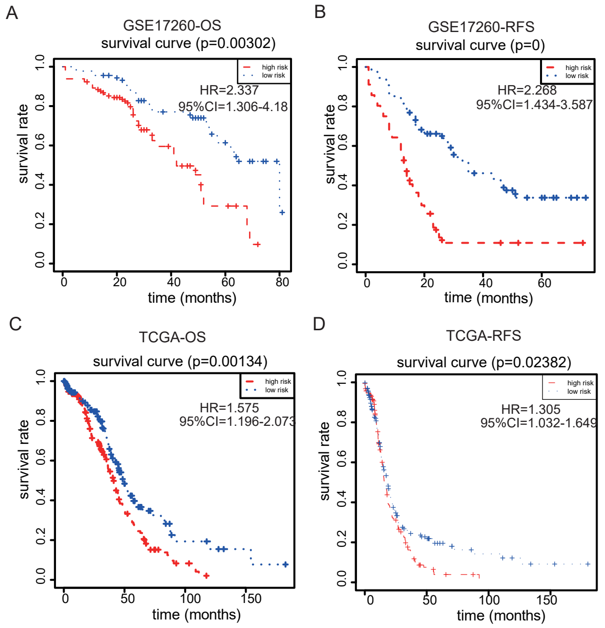
A
GSE17260-OS
survival curve (p=0.00302)
high risk
low risk
HR=2.337
95%CI=1.306-4.18
survival rate
time (months)
B
GSE17260-RFS
survival curve (p=0)
high risk
low risk
HR=2.268
95%CI=1.434-3.587
survival rate
time (months)
C
TCGA-OS
survival curve (p=0.00134)
high risk
low risk
HR=1.575
95%CI=1.196-2.073
survival rate
time (months)
D
TCGA-RFS
survival curve (p=0.02382)
high risk
low risk
HR=1.305
95%CI=1.032-1.649
survival rate
time (months)
