# Supplementary material for: Comprehensive pathway-related genes signature for prognosis and recurrence of ovarian cancer
Source: PeerJ. 2020 Dec 1;8:e10437. doi: 10.7717/peerj.10437 (PMC7718801; doi:10.7717/peerj.10437)
Supplement: Supplemental Information 5 [file peerj-08-10437-s005.docx]

Table S1. Clinical characteristics of OV in TCGA data set

| Characteristics | Entire series |
| --- | --- |
| Age(years) |  |
| >65 | 114/353 (32.3) |
| ≤65 | 239/353 (67.7) |
| Clinical stage |  |
| I | 1/353 (0.28) |
| II | 20/353 (5.67) |
| III | 274/353(77.6) |
| IV | 55/353(15.6) |
| Race |  |
| Non-white | 36/353(10.2) |
| White | 307/353(86.9) |
| Lymphatic invasion |  |
| NO | 43/353(12.2) |
| YES | 94/353(26..6) |
| Grade |  |
| G1 | 1/353(0.28) |
| G2 | 41/353(11.6) |
| G3 | 301/353(85.2) |
| G4 | 1/353(0.28) |
| Cancer status |  |
| Tumor free | 79/353(22.4) |
| With tumor | 228/353(64.6) |
