# Supplementary material for: Comprehensive pathway-related genes signature for prognosis and recurrence of ovarian cancer
Source: PeerJ. 2020 Dec 1;8:e10437. doi: 10.7717/peerj.10437 (PMC7718801; doi:10.7717/peerj.10437)
Supplement: Supplemental Information 6 [file peerj-08-10437-s006.docx]

Table S2. Brief information of GEO datasets in the study

| GEO number | Platform | Sample | |
| --- | --- | --- | --- |
|  |  | Tumor | Non-tumor |
| GSE40595 | GPL570 Affymetrix Human Genome U133 Plus 2.0 Array | 63 | 14 |
| GSE12470 | GPL887 Agilent-012097 Human 1AMicroarray (V2) G4110B | 43 | 10 |
| GSE10971 | GPL570 Affymetrix Human Genome U133 Plus 2.0 Array | 13 | 24 |
| GSE27651 | GPL570 Affymetrix Human Genome U133 Plus 2.0 Array | 43 | 6 |
| GSE38666 | GPL570 Affymetrix Human Genome U133 Plus 2.0 Array | 25 | 20 |
| GSE17260 | GPL6480 Agilent-014850 Whole Human Genome Microarray | 110 | - |
