# Supplementary material for: Comprehensive pathway-related genes signature for prognosis and recurrence of ovarian cancer
Source: PeerJ. 2020 Dec 1;8:e10437. doi: 10.7717/peerj.10437 (PMC7718801; doi:10.7717/peerj.10437)
Supplement: Supplemental Information 7 [file peerj-08-10437-s007.docx]

Table S3.Clinical characteristics of OV in GSE17260 dataset

| Characteristics | Entire series |
| --- | --- |
| Clinical stage |  |
| III | 93/110(84.5) |
| IV | 17/110(15.5) |
| Recurrence |  |
| NO | 34/110(30.9) |
| YES | 76/110(69.1) |
