# Supplementary material for: Comprehensive pathway-related genes signature for prognosis and recurrence of ovarian cancer
Source: PeerJ. 2020 Dec 1;8:e10437. doi: 10.7717/peerj.10437 (PMC7718801; doi:10.7717/peerj.10437)
Supplement: Supplemental Information 8 [file peerj-08-10437-s008.docx]

Table S4. Summary analysis for Hallmark gene sets in GEO database

| GSE40595 | Size | *P* value |
| --- | --- | --- |
| HALLMARK_MYC_TARGETS_V2 | 56 | <0.0001 |
| HALLMARK_PI3K_AKT_MTOR_SIGNALING | 104 | <0.0001 |
| HALLMARK_DNA_REPAIR | 143 | 0.0020 |
| HALLMARK_MYC_TARGETS_V1 | 189 | 0.0021 |
| HALLMARK_MTORC1_SIGNALING | 193 | 0.0244 |
| HALLMARK_OXIDATIVE_PHOSPHORYLATION | 181 | 0.0291 |
| GSE12470 | Size | *P* value |
| HALLMARK_GLYCOLYSIS | 193 | 0.0018 |
| HALLMARK_E2F_TARGETS | 188 | 0.0020 |
| HALLMARK_G2M_CHECKPOINT | 188 | 0.0114 |
| HALLMARK_DNA_REPAIR | 140 | 0.0277 |
| GSE10971 | Size | *P* value |
| HALLMARK_E2F_TARGETS | 193 | <0.0001 |
| HALLMARK_G2M_CHECKPOINT | 189 | 0.0022 |
| HALLMARK_MYC_TARGETS_V1 | 189 | 0.0066 |
| HALLMARK_GLYCOLYSIS | 195 | 0.0068 |
| HALLMARK_MYC_TARGETS_V2 | 56 | 0.0106 |
| HALLMARK_MTORC1_SIGNALING | 193 | 0.0179 |
| HALLMARK_OXIDATIVE_PHOSPHORYLATION | 181 | 0.0308 |
| HALLMARK_DNA_REPAIR | 143 | 0.0313 |
| HALLMARK_PI3K_AKT_MTOR_SIGNALING | 104 | 0.0396 |
| GSE27651 | Size | *P* value |
| HALLMARK_G2M_CHECKPOINT | 189 | 0.0150 |
| HALLMARK_E2F_TARGETS | 193 | 0.0452 |
| GSE38666 | Size | *P* value |
| HALLMARK_G2M_CHECKPOINT | 189 | <0.0001 |
| HALLMARK_E2F_TARGETS | 193 | 0.0209 |
| HALLMARK_DNA_REPAIR | 143 | 0.0213 |
