# Supplementary material for: Comprehensive pathway-related genes signature for prognosis and recurrence of ovarian cancer
Source: PeerJ. 2020 Dec 1;8:e10437. doi: 10.7717/peerj.10437 (PMC7718801; doi:10.7717/peerj.10437)
Supplement: Supplemental Information 9 [file peerj-08-10437-s009.docx]

Table S5. Pathways including LASSO genes

| Pathways | Genes |
| --- | --- |
| HALLMARK_MYC_TARGETS_V1 | SNRPA1 |
| HALLMARK_MTORC1_SIGNALING | SLC7A11, WARS |
| HALLMARK_GLYCOLYSIS | PYGB, IDUA, LHX9, DPYSL4, ISG20, |
|  | ANGPTL4, CITED2, ANGPTL4 |
| HALLMARK_G2M_CHECKPOINT | EFNA5 |
| HALLMARK_E2F_TARGETS | HMGB3 |
